# Assessment of Sequelae of COVID-19 Nearly 1 Year After Diagnosis

**DOI:** 10.3389/fmed.2021.717194

**Published:** 2021-11-23

**Authors:** Fangyuan Zhou, Meihui Tao, Luorui Shang, Yuhan Liu, Guangtao Pan, Yan Jin, Li Wang, Shaoke Hu, Jinxiao Li, Mengqi Zhang, Yu Fu, Shenglan Yang

**Affiliations:** ^1^Department of Traditional Chinese Medicine, Union Hospital, Tongji Medical College, Huazhong University of Science and Technology, Wuhan, China; ^2^Department of Gastroenterology, Union Hospital, Tongji Medical College, Huazhong University of Science and Technology, Wuhan, China; ^3^Department of Emergency, Union Hospital, Tongji Medical College, Huazhong University of Science and Technology, Wuhan, China; ^4^Department of Medical Engineering, Union Hospital, Tongji Medical College, Huazhong University of Science and Technology, Wuhan, China

**Keywords:** long-term COVID-19, SARS-CoV-2, follow-up, sequelae, multi-system assessment

## Abstract

**Background:** A previous study has shown that 81% of the COVID-19 patients had mild or moderate symptoms. However, most studies on the sequelae in COVID-19 patients focused on severe cases and the long-term follow-up studies on the health consequences in non-severe cases are limited. The current study aimed to assess the sequelae of COVID-19 in patients nearly 1 year after diagnosis with a particular focus on the recovery of patients with non-severe COVID-19.

**Methods:** We enrolled 120 patients infected with SARS-CoV-2 discharged from Wuhan Union hospital west district (designated hospital for COVID-19) and Fangcang shelter hospitals between January 29, 2020 and April 1, 2020. All participants were asked to complete a series of questionnaires to assess their symptoms and quality of life and for psychological evaluation. Also, pulmonary function test, chest CT, 6-min walking test (6MWT), routine blood test, liver and kidney function tests, fasting blood glucose test, lipid test, and immunoglobulin G antibody test were performed to evaluate their health.

**Results:** The mean age of the study population was 51.6 ± 10.8 years. Of the 120 patients, 104 (86.7%) were cases of non-severe COVID-19. The follow-up study was performed between November 23, 2020 and January 11, 2021, and the median time between the diagnosis and the follow-up was 314.5 (IQR, 296–338) days. Sleep difficulties, shortness of breath, fatigue, and joint pain were common symptoms observed during follow-up and nearly one-third of the non-severe cases had these symptoms. A total of 50 (41.7%) and 45 (37.5%) patients reported anxiety and depression, respectively. And 18.3% of the patients showed negative results in the IgG test at the follow-up, which correlated with the severity of the infection (*R* = 0.203, *p* = 0.026), and the proportion of IgG negative cases in non-severe COVID-19 patients was higher than that in the severe cases (20.2 vs. 6.3%). Pulmonary diffusion impairment was reported in 30 (26.1%) out of 115 patients, and 24 (24.2%) out of the 99 non-severe cases. The values of forced vital capacity (FVC), forced expiratory volume in 1 s (FEV1), FVC/FEV1, vital capacity (VC), total lung capacity (TLC), and residual volume (RV) were less than the normal range in 1.7, 8.6, 0.9, 11.2, 7.0, and 0.9% of the patients, respectively. A total of 55 (56.7%) out of the 97 patients showed abnormal CT findings, including ground-glass opacities (GGO), bronchiectasis, nodules, lines and bands, and fibrosis. Furthermore, there was a correlation between all the SF-36-domain scores and the duration of hospitalization, pulmonary function, and a 6MWT.

**Conclusions:** At the nearly 1-year follow-up, COVID-19 survivors still had multi-system issues, including those in the respiratory functioning, radiography, quality of life, and anxiety and depression. Moreover, non-severe cases also showed some sequelae and the proportion of IgG negative cases in the non-severe patients was higher than that in severe cases. Therefore, conducting follow-ups and preventing the reinfection of SARS-CoV-2 in this group is necessary.

## Introduction

Since December 2019, Coronavirus Disease 2019 (COVID-19) has been spreading around the world. On March 11, 2020, the World Health Organization (WHO) declared the outbreak of COVID-19 a pandemic (1). As of July 7, 2021, there were more than 180 million COVID-19 cases and over 3 million deaths worldwide, posing a tremendous burden on the health care systems (2). Despite the high mortality rate of COVID-19, most patients recovered from acute infection. Therefore, long-term follow-up studies to assess the sequelae in COVID-19 survivors are urgently needed to improve the prognosis and survival (3).

Previous studies reported that discharged COVID-19 patients had multiple health issues, such as persistent symptoms, impaired pulmonary function, chest CT abnormalities, anxiety, depression, and a decreased quality of life (4–6). The previous follow-up studies on the sequelae in COVID-19 survivors were mostly performed 1–6 months after recovery (5, 7, 8). However, a 1-year follow-up study on patients recovered from 2003 SARS showed that one-third of the patients had persistent pulmonary function impairment, and poorer health, in general, than the healthy population (9). So far, there are few follow-up studies over 1 year assessing the sequelae in the patients recovered from COVID-19 infection (10–13). Furthermore, the studies on multi-system assessment of long-term consequences in COVID-19 survivors are limited. A previous study reported that 81% of the patients with COVID-19 showed mild to moderate disease (14). However, most studies focused on the sequelae in severe cases, and the data on non-severe cases is limited, especially on those treated at the Fangcang Hospital (5, 15). In the current study, we aimed to evaluate the health status of COVID-19 patients nearly 1 year after diagnosis, with a particular focus on the recovery of patients with non-severe COVID-19.

## Methods

### Study Design and Participants

In the current cohort study, we enrolled laboratory-confirmed COVID-19 patients discharged from the Wuhan Union hospital west district (designated hospital for COVID-19) and Fangcang shelter hospitals between January 29, 2020 and April 1, 2020. After excluding patients without considerable data due to physiological or subjective rejection tests, data on 120 patients were considered for the analyses. The patients were classified as severe based on the guidelines for the severity of infection provided in the “Diagnosis and Treatment Protocol for Novel Coronavirus Pneumonia” issued by the National Health Commission, which were—shortness of breath, respiratory rate (RR) ≥ 30 /min, oxygen saturation ≤ 93% at rest, and arterial oxygen pressure (PaO_2_)/fraction of inspiration of O_2_ (FiO_2_) ≤ 300 mm Hg; and the rest of the patients were considered as non-severe cases (16). All enrolled patients met the discharge standards as indicated in the Diagnosis and Treatment Protocol issued by the National Health Commission, including no fever for three consecutive days, obvious improvement in the respiratory symptoms, acute lesions on imaging, and two consecutive negative results of the SARS-CoV-2 RNA tested using real-time reverse-transcriptase-polymerase-chain-reaction (RT-PCR) with an interval of 24 h.

The study was approved by the ethics committee of the Wuhan Union Hospital (UHCT-IEC-SOP-016-02-01) and the Chinese Clinical Trial Registry (ChiCTR2100049283). We obtained written informed consent from all the participants during enrollment.

### Follow-Up Assessment

Follow-up was performed nearly 1 year after being diagnosed with COVID-19. All patients were asked to complete a series of questionnaires to assess their symptoms and quality of life and for psychological evaluation. The modified British Medical Research Council (mMRC) dyspnea scale and Brog scale were used to assess self-reported dyspnea (17). Anxiety and depression were measured using the Hamilton Anxiety Scale (HAMA) and Hamilton Depression Scale (HAMD), respectively (18, 19). The MOS item short-form health survey (SF-36), which assessed the Physical Functioning (PF), Role-Physical (RP), Bodily Pain (BP), General Health (GH), Vitality (VT), Social Functioning (SF), Role-Emotional (RE), and Mental Health (MH), was used to evaluate the Health-Related Quality of Life (HRQoL) (20). Furthermore, the patients underwent a series of laboratory examinations to evaluate their health, including routine blood test, liver and kidney function tests, fasting blood glucose test, lipid test, and immunoglobulin (Ig) G antibody test. The IgG antibodies against the nucleoprotein of SARS-CoV-2 were measured using enzyme-linked immunosorbent assay (ELISA) kits (Beijing Wantai Biological Pharmacy Enterprise Co., Ltd.) as reported previously (21).

### Pulmonary Function Test

The pulmonary function test was performed in the Lung Function Laboratory of the Wuhan Union Hospital according to the current guidelines by the American Thoracic Society (22). Pulmonary function parameters included FVC, FEV1, FEV1/FVC, VC, TLC, RV, Diffusing capacity for carbon monoxide (DLCO), and DLCO/VA. In the light of the guidelines by the European Community Lung Health Survey, the results are shown as a percentage of the predicted value (23). The 6MWT was performed based on the guidelines by the American Thoracic Society (24). The blood oxygen saturation of the participants was also measured before and after the 6MWT.

### Radiographic Assessment

The radiographic images were evaluated by experienced radiologists who were blinded to the clinical data. We assessed 97 patients' chest CT images for the presence of ground-glass opacities, bronchiectasis, nodules, lines and bands, and fibrosis. The percentage of involvement of each of the five lobes was transformed into a corresponding score as following −0, no involvement; 1, 1–25% involvement; 2, 26–50% involvement; 3, 51–75% involvement; and 4, 75–100% involvement. The “total severity score (TSS)” was the sum of the scores of the five lobes (range 0–20) (25). In addition, the artificial intelligence software (YT-CT-Lung, YITU Healthcare Technology Co., Ltd., China) was used to quantify and analyze the chest CT images of the patients (26).

### Statistical Analysis

Continuous variables are expressed as means (standard deviations, SD) or medians (interquartile ranges, IQR). The normality of the distribution was estimated using the Kolmogorov–Smirnov test. Categorical variables were described as numbers (percentages, %). We appropriately adopted the *t*-test, Mann–Whitney *U*-test, χ^2^ test, and Fisher's exact test to compare the characteristics of the COVID-19 survivors of different severities according to variable types and distribution characteristics. The correlations of SF-36 scores across the eight domains with the length of hospitalization, 6-min walking distance (6MWD), and pulmonary function were measured using Spearman's correlation test. All statistical analyses were performed using SPSS (Statistical Package for the Social Sciences) version 21.0. Statistical tests were two-tailed, and a *P* < 0.05 was considered statistically significant.

## Results

### Characteristics of Enrolled COVID-19 Patients

The clinical characteristics of 120 participants are shown in Table 1. There were 104 (86.7%) non-severe cases and 16 (13.3%) severe cases. The mean age was 51.6 (±10.8) years, and 49 (40.8%) of them were men. A total of 16 (13.3%) patients had a history of smoking. The most common comorbidities observed were hypertension (20 [16.7%]), followed by diabetes (6 [5%]). Compared to non-severe cases, diabetes accounted for a larger proportion in severe cases (*p* = 0.031). The median duration of hospital stay was 25.5 (IQR, 18–33.8) days and the median time from discharge to follow-up was 284.5 (270–309) days. The median time from diagnosis to follow-up was 314.5 (296–338) days, ranging from 274 to 379 days.

**Table 1:** Demographic and clinical characteristics of the enrolled COVID-19 patients.

	**Total (*N* = 120)**	**Non-severe (*n* = 104)**	**Severe (*n* = 16)**	***P*-value**
**Demographic characteristics**
Age, years	51.6 ± 10.8	51.4 ± 10.9	52.6 ± 10.1	0.67
**Sex**
Men	49 (40.8)	41 (39.4)	8 (50)	0.423
Women	71 (59.2)	63 (60.6)	8 (50)	
**Cigarette smoking**
Yes	16 (13.3)	14 (13.5)	2 (12.5)	1
No	104 (86.7)	90 (86.5)	14 (87.5)	
**Comorbidities**
Diabetes	6 (5)	3 (2.9)	3 (18.8)	0.031
Hypertension	20 (16.7)	16 (15.4)	4 (25)	0.548
Hyperlipidemia	4 (3.3)	3 (2.9)	1 (6.3)	0.44
Coronary heart disease	3 (2.5)	3 (2.9)	0 (0)	1
Length of hospitalization, days	25.5 (18–33.8)	24.5 (18–32.8)	31 (17.5–41.5)	0.114
Days from diagnosis to follow-up, days	314.5 (296–338)	315.5 (296–338.8)	307.5 (296.3–327.3)	0.671
Days from discharge to follow-up, days	284.5 (270–309)	288.5 (270.5–309.8)	274.5 (256–294)	0.066

*Data are presented as mean ± SD, median (interquartile range), and n (%)*.

*Continuous variables were tested using the t-test, while categorical variables were compared using the χ^2^ test or Fisher's exact test*.

### Symptoms and the Results of the Laboratory Tests at the Nearly 1-Year Follow-Up

Table 2 shows the body mass index (BMI), symptoms, and the results of the laboratory tests during follow-up. The mean BMI was 24.6 (± 2.8) kg/m^2^, and the severe cases showed a significantly higher BMI than the non-severe ones (*p* = 0.02). The most common symptoms in COVID-19 survivors at the nearly 1-year follow-up were sleep difficulties (52 [43.3%]), shortness of breath (49 [40.8%]), fatigue (43 [35.8%]), and joint pain (39 [32.5%]), and nearly one-third of the non-severe cases had these symptoms. At follow-up, all participants showed healthy levels of white blood cells, lymphocytes, hemoglobin, and platelets. In addition, the proportions of patients with levels of alanine aminotransferase (ALT), aspartate aminotransferase (AST), creatinine, triglyceride, total cholesterol, and fasting blood glucose above the normal range were 21/119 (17.6%), 8/119 (6.7%), 20/119 (16.8%), 51/119 (42.9%), 71/119 (59.7%), and 20/118 (16.9%), respectively. Compared to the non-severe cases, severe cases showed significantly higher lymphocytes (*p* = 0.041), hemoglobin (*p* = 0.013), ALT (*p* = 0.011), triglyceride (*p* = 0.049), and fasting blood glucose (*p* = 0.033). The results of the IgG test were negative in 22 (18.3%) patients, and the proportion of IgG negative patients in non-severe cases was higher than that in severe cases (20.2 vs. 6.3%), but the difference was not statistically significant (*p* = 0.075). At the nearly 1-year follow-up, IgG test results correlated with the severity of the disease (*R* = 0.203, *p* = 0.026).

**Table 2:** Symptoms, laboratory findings, and mental and cognitive status at the nearly 1-year follow-up.

	**Total (*N* = 120)**	**Non-severe (*n* = 104)**	**Severe (*n* = 16)**	***P*-value**
BMI, kg/m^2^	24.6 ± 2.8	24.3 ± 2.8	26.1 ± 2.9	0.02
**Symptoms**
Shortness of breath	49 (40.8)	40 (38.5)	9 (56.3)	0.178
Fatigue	43 (35.8)	34 (32.7)	9 (56.3)	0.067
Sleep difficulties	52 (43.3)	44 (42.3)	8 (50)	0.563
Joint pain	39 (32.5)	33 (31.7)	6 (37.5)	0.646
Loss of Smell	9 (7.5)	7 (6.7)	2 (12.5)	0.76
Constipation	21 (17.5)	18 (17.3)	3 (18.8)	1
Diarrhea	4 (3.3)	3 (2.9)	1 (6.3)	0.44
**Laboratory findings**
White blood cell count, ×10^9^/L	5.9 ± 1.6	5.8 ± 1.6	6.5 ± 1.6	0.16
Lymphocytes count, ×10^9^/L	1.9 ± 0.5	1.8 ± 0.4	2.1 ± 0.5	0.041
Hemoglobin, g/L	146.8 ± 15.8	145.3 ± 16.1	156.1 ± 10.2	0.013
Platelet count, ×10^9^/L	221.7 ± 59.9	220.4 ± 58.7	229.5 ± 68.6	0.586
ALT, U/L	23 (18–31)	22 (17–30)	28.5 (25–66.3)	0.011
>35	21/119 (17.6)	16 (15.5)	5 (31.3)	0.237
AST, U/L	23 (20–29)	23 (19–29)	26 (22.3–29.5)	0.178
>40	8/119 (6.7)	5 (4.9)	3 (18.8)	0.126
Creatinine, μmol/L	62.4 ± 15.4	62.5 ± 15.9	61 ± 11.7	0.627
>81	20/119 (16.8)	19 (18.4)	1 (6.3)	0.225
Triglyceride, mmol/L	1.8 ± 0.9	1.7 ± 0.9	2.2 ± 1	0.049
>1.7	51/119 (42.9)	42 (40.8)	9 (56.3)	0.245
Total cholesterol, mmol/L	5.5 ± 0.9	5.4 ± 0.9	5.7 ± 1	0.146
>5.2	71/119 (59.7)	61 (59.2)	10 (62.5)	0.804
Fasting blood glucose, mmol/L	5.5 (5.1–5.9)	5.5 (5.1–5.9)	5.9 (5.5–6.2)	0.033
>6.1	20/118 (16.9)	16 (15.7)	4 (25)	0.572
**IgG**
Negative	22 (18.3)	21 (20.2)	1 (6.3)	0.075
Weakly positive	32 (26.7)	30 (28.8)	2 (12.5)	
Positive	66 (55)	53 (51)	13 (81.3)	
**Mental status**
HAMA	5 (2–11.5)	5 (2–9)	7.5 (1.5–13)	0.496
0–6 (no)	70 (58.3)	63 (60.6)	7 (43.8)	0.317
7–13 (mild/moderate)	29 (24.2)	23 (22.1)	6 (37.5)	
≥14 (severe/extreme)	21 (17.5)	18 (17.3)	3 (18.8)	
HAMD	4.0 (2–9)	4 (2–9)	6 (3.3–10.8)	0.405
0–6 (no)	75 (62.5)	65 (62.5)	10 (62.5)	0.817
7–23 (mild/moderate)	39 (32.5)	33 (31.7)	6 (37.5)	
≥24 (severe/extreme)	6 (5)	6 (5.8)	0 (0)	

*Data are presented as mean ± SD, median (interquartile range), and n (%)*.

*Continuous variables were tested using the t-test or Mann–Whitney U-test, while categorical variables were compared using the χ^2^ test or Fisher's exact test*.

*BMI, body mass index; ALT, alanine aminotransferase; AST, aspartate aminotransferase; HAMA, Hamilton Anxiety Scale; HAMD, Hamilton Depression Scale*.

### Results of the Psychological Assessment and Quality of Life at the Nearly 1-Year Follow-Up

**Figure 1:**
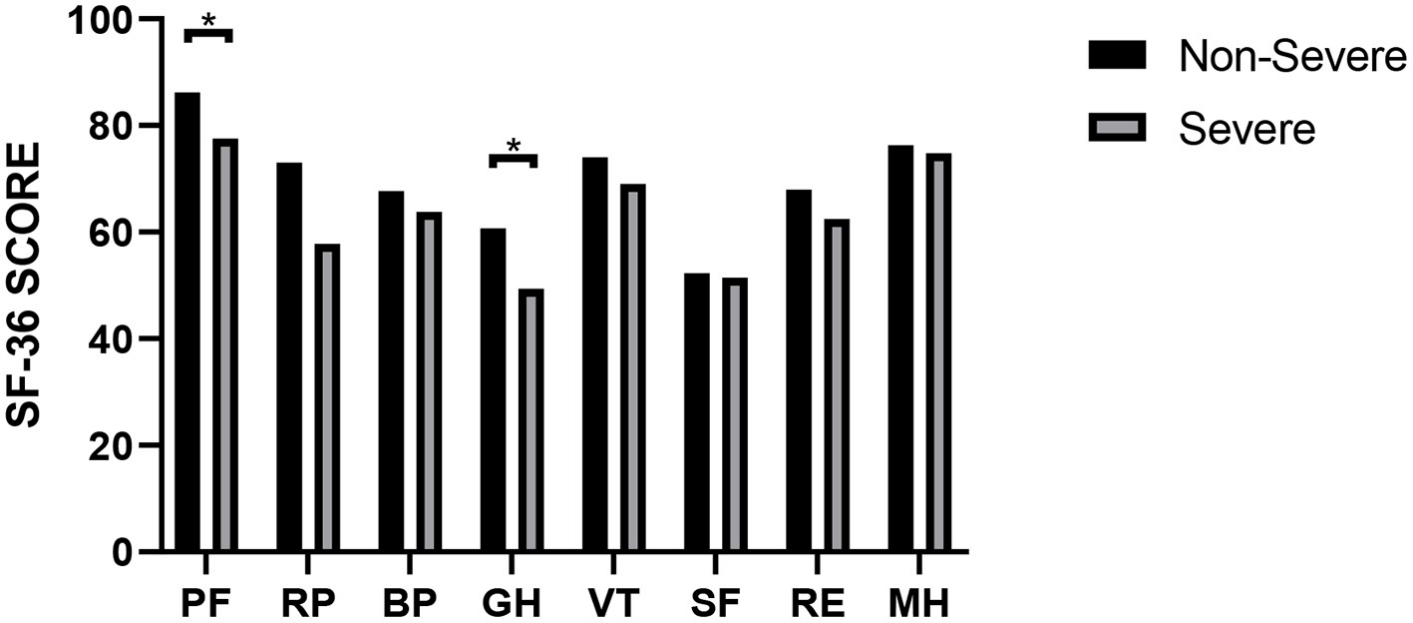
SF-36 scores of COVID-19 patients with different severities. *P < 0.05. *P*-values were calculated using the *t*-test.

At the nearly 1-year follow-up, 50 (41.7%) and 45 (37.5%) patients showed greater-than-normal HAMA and HAMD scores, respectively, while in the non-severe cases, 41 (39.4%) reported anxiety and 39 (37.5%) reported depression (Table 2). Figure 1 shows the average scores across the 8 domains of SF-36 used for assessing HRQoL. All SF-36-domain scores significantly decreased. Patients with the severe disease had significantly lower levels of PF and GH than the non-severe cases (*p* = 0.049 and 0.045, respectively). Table 3 shows the correlations between all the SF-36-domain scores and the length of hospitalization, pulmonary function, and the 6MWD. Except for SF, other domains of SF-36 negatively correlated with length of hospitalization, among which the correlations with RP and GH were statistically significant. FEV1/FVC, DLCO, and FEV1 showed significant positive correlations with PF, RP, and GH, respectively. Furthermore, 6MWD was positively associated with PF, GH, VT, and RE.

**Table 3:** Correlations among HRQoL, length of hospitalization, pulmonary function, and 6MWD at the nearly 1-year follow-up.

**SF-36**	**LOH**	**FVC**	**FEV1**	**FEV1/FVC**	**VC**	**TLC**	**DLCO**	**6MWD**
PF	−0.16	0.03	0.11	0.19^*^	0.01	0.07	0.16	0.22^*^
RP	−0.25^†^	0.16	0.16	0.02	0.14	0.14	0.219^*^	0.15
BP	−0.14	0.04	0.06	0.08	0.05	0.07	−0.01	−0.11
GH	−0.18^*^	0.1	0.21^*^	0.15	0.07	0.1	0.15	0.2^*^
VT	−0.16	−0.04	0.05	0.22^*^	−0.06	−0.03	0.12	0.22^*^
SF	0.02	−0.02	0.02	0.08	0	0.02	0.15	0.08
RE	−0.01	0.15	0.17	0.09	0.12	0.09	0.14	0.21^*^
MH	−0.14	−0.01	0.07	0.17	−0.04	−0.03	0.03	0.14

*Values shown are Spearman correlation coefficients (r)*.

* *P <0.05*;

† *P <0.01*.

*LOH, length of hospitalization; 6MWD, 6-minute walk distance; PF, Physical Functioning; RP, Role-Physical; BP, Bodily Pain; GH, General Health; VT, Vitality; SF, Social Functioning; RE, Role-Emotional; MH, Mental Health*.

### Results of the Pulmonary Function Test, 6MWT, and Chest CT Assessment at the Nearly 1-Year Follow-Up

As shown in Table 4, the proportions of mMRC ≥ 1 and Brog ≥ 1 were significantly higher in the severe cases than the non-severe cases, and these proportions were 32.7 and 34.6% in the non-severe cases, respectively. The mean 6MWD of participants was 514.1 (± 73) m, and there was no significant decrease in the oxygen saturation after the walk. The walk distance was below 80% of the predicted value in 18.5% of the total patients and 18.4% of the non-severe cases. Pulmonary diffusion impairment was found in 30/115 (26.1%) participants and 24 (24.2%) of the 99 non-severe cases. The proportions of patients with FVC, FEV1, FVC/FEV1, VC, TLC, and RV were lower than the lower limit of the normal range were 2/116 (1.7%), 10/116 (8.6%), 1/116 (0.9%), 13/116 (11.2%), 8/115 (7.0%), and 1/114 (0.9%), respectively. The differences in FVC, FEV1, VC, TLC, RV, and DLCO/VA were significant between the severe and non-severe cases. Follow-up chest CT evaluation showed that 55/97 (56.7%) patients showed abnormal CT findings, including 47 (56.6%) non-severe cases and 8 (57.1%) severe cases. The most common abnormal CT manifestations were nodules (55.7%), followed by lines and bands (47.4%), fibrosis (17.5%), GGO (16.5%), and bronchiectasis (14.4%). TSS ≥ 1 was found in 15/97 (15.5%) patients, and the maximum TSS score was 13. Besides, quantitative assessment of CT showed that the volume of the residual lesions, GGO, and consolidation of bilateral lung were 0.03 (0–0.58) cm^3^, 0.02 (0–0.45) cm^3^, and 0.01 (0–0.07) cm^3^, respectively.

**Table 4:** Dyspnea, pulmonary function, 6MWD, and chest CT assessment at the nearly 1-year follow-up.

	**Total (*N* = 120)**	**Non-severe (*n* = 104)**	**Severe (*n* = 16)**	***P*-value**
**Dyspnea**
***mMRC score***
≥1	46 (38.3)	34 (32.7)	12 (75)	0.001
***Brog score***
≥1	47 (39.2)	36 (34.6)	11 (68.8)	0.009
**Pulmonary function**
FVC, % of predicted	108.6 ± 17.5	110 ± 17.7	100.2 ± 14.4	0.037
FVC <80%, No. (%)	2/116 (1.7)	1/100 (1)	1/16 (6.3)	0.258
FEV1, % of predicted	99.8 ± 13.5	101 ± 13.3	92.3 ± 12.7	0.016
FEV1 <80%, No. (%)	10/116 (8.6)	6/100 (6)	4/16 (25)	0.042
FEV1/FVC, % of predicted	92 ± 7.4	91.6 ± 7.4	94.3 ± 7.2	0.186
FEV1/FVC <70%, No. (%)	1/116 (0.9)	1/100 (1)	0/16 (0)	1
VC, % of predicted	107.1 ± 14.5	108.5 ± 14.3	98.7 ± 13.6	0.012
VC <90%, No. (%)	13/116 (11.2)	9/100 (9)	4/16 (25)	0.145
TLC, % of predicted	95.1 ± 10.8	96.2 ± 10.2	88 ± 12.5	0.005
TLC <80%, No. (%)	8/115 (7.0)	4/99 (4)	4/16 (25)	0.011
RV, % of predicted	90.2 ± 13.6	91.6 ± 13.3	81.9 ± 12.4	0.007
RV <60%, No. (%)	1/114 (0.9)	0/98 (0)	1/16 (6.3)	0.14
DLCO, % of predicted	88.4 ± 12.5	88.6 ± 12.5	87.3 ± 12.7	0.697
DLCO <80%, No. (%)	30/115 (26.1)	24/99 (24.2)	6/16 (37.5)	0.416
DLCO/VA, % of predicted	96.2 ± 13.7	95 ± 13.6	103.1 ± 12.1	0.027
**6MWD**
Distance, m	514.1 ± 73	517.3 ± 72.2	493.5 ± 77.3	0.225
% of predicted	92.4 (84.1–101.1)	92.6 (84.4–103.1)	91 (81.3–97.6)	0.445
<80% predicted value, No. (%)	22/119 (18.5)	19/103 (18.4)	3/16 (18.8)	1
SpO_2_ before exercise, %	98 (97–99)	98 (97–99)	97 (97–98.8)	0.029
SpO_2_ after exercise, %	98 (98–99)	99 (98–99)	98 (97–99)	0.103
**Chest CT**
**Abnormal CT findings, No. (%)**	55/97 (56.7)	47/83 (56.6)	8/14 (57.1)	0.971
**TSS**	0 (0–0)	0 (0–0)	0 (0–0.5)	0.535
≥1, No. (%)	15/97 (15.5)	12/83 (14.5)	3/14 (21.4)	0.789
**Type of chest CT abnormalities present, No. (%)**
GGO	16/97 (16.5)	11/83 (13.3)	5/14 (35.7)	0.088
Bronchiectasis	14/97 (14.4)	13/83 (15.7)	1/14 (7.1)	0.738
nodules	54/97 (55.7)	47/83 (56.6)	7/14 (50)	0.644
Lines and bands	46/97 (47.4)	41/83 (49.4)	5/14 (35.7)	0.343
Fibrosis	17/97 (17.5)	14/83 (16.9)	3/14 (21.4)	0.972
**Quantitative assessment**
Volume of bilateral pulmonary lesions (cm^3^)	0.03 (0–0.58)	0.03 (0–0.6)	0.03 (0–0.73)	0.81
Volume of bilateral pulmonary GGO lesions (cm^3^)	0.02 (0–0.45)	0.01 (0–0.54)	0.03 (0–0.67)	0.896
Volume of bilateral pulmonary consolidation lesions (cm^3^)	0.01 (0–0.07)	0.01 (0–0.07)	0.01 (0–0.06)	0.597
Percent of bilateral pulmonary lesions (%)	0 (0–0.01)	0 (0–0.01)	0 (0–0.02)	0.763
Percent of bilateral pulmonary GGO lesions (%)	0 (0–0.01)	0 (0–0.01)	0 (0–0.02)	0.99
Percent of bilateral pulmonary consolidation lesions (%)	0 (0–0)	0 (0–0)	0 (0–0)	0.1

*Data are presented as mean ± SD, median (interquartile range), and n (%). Continuous variables were tested using the t-test or Mann–Whitney U-test, while categorical variables were compared using the χ^2^ test or Fisher's exact test. mMRC, modified British Medical Research Council; 6MWD, 6-minute walk distance; TSS, total severity score; GGO, ground-glass opacities*.

## Discussion

To the best of our knowledge, to date, there are few studies assessing the health outcomes of COVID-19 patients 1 year after diagnosis (10–13). In the current cohort study, we evaluated the health status of COVID-19 survivors nearly 1 year after diagnosis. We found that participants showed certain symptoms like anxiety, depression, and impaired HRQoL, as well as residual chest CT abnormalities, accompanied by pulmonary diffusion impairment. Also, the levels of the indicators for liver and kidney functions were found to be higher than the normal range.

In the current study, we found that COVID-19 survivors showed sleep difficulties, shortness of breath, fatigue, and abnormal anxiety and depression at the nearly 1-year follow-up, and more than one-third of the non-severe cases showed these symptoms. These observations were consistent with those of the previous studies. It was shown that several COVID-19 survivors reported fatigue, sleep difficulties, anxiety, and depression 6 months after the onset of the illness (5). A 4-year follow-up study of 2003 SARS reported that 40% of the survivors had persistent fatigue and psychological sequelae (27). In addition, participants suffered from impaired HRQoL, as assessed using the Chinese version of the SF-36 questionnaire. These psychological sequelae and impaired HRQoL may be associated with a variety of factors, including direct lung injury and neurological involvement caused by the viral infection, the long period of isolation, and the anxiety caused by the pandemic (28).

Our study showed that more than one-third of the subjects reported abnormal mMRC and Brog scores. Some participants had impaired pulmonary function during follow-up, the most common of which was DLCO abnormalities (26.1%). This was consistent with another follow-up study on 2003 SARS, where 23.7% of the survivors developed diffusing-capacity impairment 1 year after the onset of the symptoms (29). We found that a considerable proportion (56.7%) of the patients showed abnormalities in their corresponding radiographic images, and the major findings were nodules, lines and bands, and fibrosis. Previous studies showed interstitial changes in the lungs as the most common long-term pulmonary abnormality in COVID-19 and 2003 SARS survivors (5, 30). It was shown that even at 12 months after discharge, 24% of the COVID-19 survivors had persistent abnormalities in their radiographical images, which included GGO, interlobular septal thickening, reticular opacity, and mosaic attenuation (10). The proportions of residual pulmonary abnormalities were higher in our study, which may be due to the use of artificial intelligence for the evaluation and detection of more subtle changes. Moreover, compared to the residual lung lesion volume in a previous study performed 6 months after the onset of the symptoms, the volume of bilateral pulmonary lesions was significantly lesser in our study (5). It suggested that although some patients showed abnormalities in their radiographic images, the intensity of impairment was small and the lesions were continuously resolved. A 15-year follow-up study showed that the interstitial changes in the lungs and the decline in the lung function caused by 2003 SARS may be successfully treated, and significant recovery can be achieved within 2 years (31). Whether recovery in pulmonary function and abnormalities observed in the radiographic images is possible remains to be further investigated.

The inflammatory indicators in the current study were within the normal range but abnormal ALT, AST, and creatinine values were observed in a few COVID-19 survivors. As reported previously, a proportion of COVID-19 survivors showed abnormal liver function test results during follow-up (32). Furthermore, 35% of the COVID-19 survivors had an eGFR <90 mL/min per 1.73 m^2^ but 13% of these patients presented normal eGFR at the acute phase (5). This indicated that abnormal indicators in COVID-19 survivors were not necessarily a persistent impairment after acute injury. This may also be caused by factors during the post-acute phase, such as drugs and fatty liver caused by overnutrition and other factors. However, since the liver and kidney function test results of the subjects before viral infection were unavailable, the liver and kidney function abnormalities could not be completely attributed to COVID-19 and long-term follow-up studies are necessary. Besides, 18.3% of the patients had negative results in the IgG test, and a higher proportion of the non-severe cases showed negative results in the IgG test. There was a correlation between IgG conversion and the severity of infection. A study of COVID-19 immune response 8 weeks after discharge found that the proportions of IgG negative patients in the asymptomatic and symptomatic cases were 40 and 12.9%, respectively (33). As reported previously, patients with severe disease had higher levels of IgG than asymptomatic individuals and mild cases (34), suggesting that the severity of the disease is associated with the immune response to the viral infection. A previous study on 2003 SARS survivors demonstrated that IgG was still detectable in the patients 16 months after the onset of the illness (35). Hence, the immune response to SARS-CoV-2 needs to be investigated in further long-term studies.

There were some limitations to the current study. Firstly, it was a small cohort study. We did not study the longitudinal physical and psychological changes in the COVID-19 survivors with a prolonged recovery time. In the future, we will continue to follow the COVID-19 patients and observe changes in the sequelae. Secondly, our study sampled a small population. However, our results provided further insights into the understanding of COVID-19. Larger sample size is needed to investigate the sequelae in COVID-19 patients with differing severities. Thirdly, the baseline data before the SARS-CoV-2 infection and during the acute phase were not available. Therefore, the abnormal health status of COVID-19 survivors cannot be attributed exclusively to the viral infection. However, although it may be underestimated since it was self-reported by the patients, the proportion of patients with underlying diseases was low. Finally, due to the lack of a control group that had not contracted SARS-CoV-2, the sequelae of COVID-19 could not be better evaluated.

In conclusion, the discharged patients suffered from multi-system issues nearly 1 year after being diagnosed with COVID-19, even the non-severe cases. Moreover, the proportion of IgG negative cases was higher in the non-severe cases than the severe ones. Our results complemented the current limitations of research on the long-term effects in COVID-19 patients with the non-severe disease and enabled us to have a more comprehensive understanding of COVID-19. The results suggested that physicians should also pay attention to post-discharge care in non-severe cases, and the non-severe cases should also be studied to fully understand the health consequences after viral infection and prevent the reinfection of SARS-CoV-2.

## Data Availability Statement

The raw data supporting the conclusions of this article will be made available by the authors, without undue reservation.

## Ethics Statement

The study was approved by the Ethics Committee of the Wuhan Union Hospital. Written informed consent was obtained from all the participants during enrollment.

## Author Contributions

SY conceptualized and designed the study and had full access to all of the data in the study. SY and YF took responsibility for the integrity of the data and the accuracy of the data analysis. LS, YL, MZ, GP, and JL summarized the data. FZ and MT contributed to data analysis and drafted the manuscript. SH contributed to data platform establishment and artificial intelligence analysis. LW took the responsibility of project contact. YJ was responsible for patient care and communication. All authors contributed to data acquisition or data interpretation, reviewed the manuscript, and approved the final version.

## Funding

This study was funded by Key R&D Program of Hubei Province (Grant Number: 2020BCA065).

## Conflict of Interest

The authors declare that the research was conducted in the absence of any commercial or financial relationships that could be construed as a potential conflict of interest.

## Publisher's Note

All claims expressed in this article are solely those of the authors and do not necessarily represent those of their affiliated organizations, or those of the publisher, the editors and the reviewers. Any product that may be evaluated in this article, or claim that may be made by its manufacturer, is not guaranteed or endorsed by the publisher.
